# Modeling atrial fibrosis *in vitro*—Generation and characterization of a novel human atrial fibroblast cell line

**DOI:** 10.1002/2211-5463.12896

**Published:** 2020-06-02

**Authors:** Stephan R. Künzel, Johanna S. E. Rausch, Charlotte Schäffer, Maximilian Hoffmann, Karolina Künzel, Erik Klapproth, Theresa Kant, Natalie Herzog, Jan‐Heiner Küpper, Kristina Lorenz, Svenja Dudek, Ramona Emig, Ursula Ravens, Eva A. Rog‐Zielinska, Rémi Peyronnet, Ali El‐Armouche

**Affiliations:** ^1^ Institute of Pharmacology and Toxicology Faculty of Medicine Carl Gustav Carus Technische Universität Dresden Dresden Germany; ^2^ Institute of Biotechnology Brandenburg University of Technology Cottbus‐Senftenberg Senftenberg Germany; ^3^ Institute of Pharmacology and Toxicology University of Würzburg Würzburg Germany; ^4^ Leibniz‐Institut für Analytische Wissenschaften – ISAS e. V. Dortmund Germany; ^5^ Institut für Experimentelle Kardiovaskuläre Medizin Universitäts Herzzentrum, Freiburg Bad ‐ Krozingen Freiburg im Breisgau Germany; ^6^ Faculty of Medicine University of Freiburg Freiburg Germany

**Keywords:** cardiovascular disease, cell culture, cell lines, fibroblasts, fibrosis, heart

## Abstract

Atrial fibrillation (AF) is regularly accompanied by cardiac fibrosis and concomitant heart failure. Due to the heterogeneous nature and complexity of fibrosis, the knowledge about the underlying mechanisms is limited, which prevents effective pharmacotherapy. A deeper understanding of cardiac fibroblasts is essential to meet this need. We previously described phenotypic and functional differences between atrial fibroblasts from patients in sinus rhythm and with AF. Herein, we established and characterized a novel human atrial fibroblast line, which displays typical fibroblast morphology and function comparable to primary cells but with improved proliferation capacity and low spontaneous myofibroblast differentiation. These traits make our model suitable for the study of fibrosis mechanisms and for drug screening aimed at developing effective antifibrotic pharmacotherapy.

## Graphical abstract

Using lentiviral transfer of Upcyte^®^ proliferation genes, a novel human atrial fibroblast cell line (HAF‐SRK01) was generated. Proliferation was significantly increased and spontaneous myofibroblast differentiation significantly decreased compared to primary cells. Functional analyzes confirmed that the cell line is suitable for recapitulating fibrosis development. These traits predispose the model for antifibrotic drug screening and to study fibrosis mechanisms.

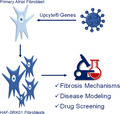

**Abbreviations**
AF: atrial fibrillation
CVD: cardiovascular diseases
ECM: extracellular matrix
HAF: human atrial fibroblast
HVF: human ventricular fibroblast
PAF: primary atrial fibroblast
SR: sinus rhythm

Cardiovascular diseases (CVD) such as heart failure and arrhythmia represent the leading cause of death worldwide [1, 2]. A well‐recognized concomitant of CVD is cardiac fibrosis [1], which is the structural manifestation of an imbalance in extracellular matrix (ECM) homeostasis. With nearly 45% of all deaths in the Western world attributable to fibroproliferative disease, the clinical relevance of fibrotic remodeling is enormous [3, 4]. This is particularly true in the case of atrial fibrosis, associated with a detrimental clinical outcome of highly abundant supraventricular arrhythmias like atrial fibrillation (AF) [5]. However, due to the heterogeneous nature and complexity of the pro‐fibrotic processes, the mechanistic knowledge is limited. For this reason, effective antifibrotic pharmacotherapy is still elusive [6].

The key cellular mediators of cardiac fibrosis are fibroblasts [7, 8]. In the healthy heart, fibroblasts regulate ECM homeostasis by secretion and degradation of ECM components thereby safeguarding the mechanical integrity of the myocardium. Furthermore, fibroblasts have been shown to play regulatory roles in cardiac electrophysiology and inflammation processes [9]. Upon activation by pathological stimuli like myocardial injury or arrhythmia, fibroblasts undergo a phenotypical and functional transition toward myofibroblasts [10]. Once activated, myofibroblasts increase in size, commence expression of orderly arranged filaments of alpha‐smooth muscle actin (αSMA), and become highly secretory, resulting in enhanced deposition of interstitial collagen as well as local enrichment of cytokines and other mediators of inflammation [11]. Although this process is initially beneficial for wound healing, a prolonged presence of myofibroblasts is detrimental for physiological cardiac function [12].

Fibroblast cultures have become invaluable models for the study of fibrosis mechanisms *in vitro* [13] for derivation of novel targeted therapy strategies. However, human atrial fibroblast (HAF) cell lines are scarcely available and primary human fibroblasts are difficult to obtain on a regular basis for medical and ethical reasons. Thus, the objectives of our study were (a) to generate and to characterize a new model of HAF s to investigate atrial fibrosis mechanisms and (b) to highlight the intracardial phenotypic heterogeneity between atrial and ventricular fibroblast models.

## Materials and methods

### Informed consent

All patients participating in this study gave written informed consent according to the Declaration of Helsinki (file number of the institutional review committee: EK 114082202). The collection and study of human samples in Freiburg were reviewed and approved by the Ethics Commission of the University of Freiburg, Freiburg, Germany (reference: 393/16: 214/18).

### Cell acquisition and culture conditions

An immortalized human ventricular fibroblast cell line (HVF) was purchased from ABM Inc. (Richmond, BC, Canada). Cardiac right atrial tissue biopsies were obtained during open‐heart surgery in cooperation with Herzzentrum Dresden GmbH. Primary HAFs (PAF) were isolated from the tissue biopsies *via* outgrowth as described previously [10]. Fibroblasts were cultured on noncoated NUNC cell culture flasks in DMEM (10% FCS, 1% penicillin–streptomycin) at 37 °C and 5% CO_2_.

### Lentiviral transfer of proliferation genes

By lentiviral transfer of Upcyte® proliferation genes [14, 15] (Invitrogen, Karlsruhe, Germany) into primary atrial fibroblasts (PAF) of a male donor, we generated the nontransformed HAF cell line (HAF‐SRK01) with extended lifespan and proliferation competence. The donor was selected among available candidates based on adequate health (see Table 1 for donor characteristics).

**Table 1:** Donor characteristics.

Item	Value
Age (Years)	83
Sex	Male
BMI (kg·m^−2^)	30.8
Grade of heart failure	NYHA 2
Ejection fraction (%)	60
Hypertension	Yes
Diabetes mellitus	No

### Functional fibroblast characterization

Experiments to determine fibroblast proliferation, spontaneous myofibroblast differentiation, and wound healing capacity (migration) were performed as described previously [10].

#### Proliferation

Cells were seeded on 12‐well plates at densities of 1 × 10^4^/ well. Cells were harvested and counted after 7 and 14 days using 0.25 % trypsin and a Burker counting chamber.

#### Myofibroblast differentiation and immunocytochemistry

**Fig. 1:**
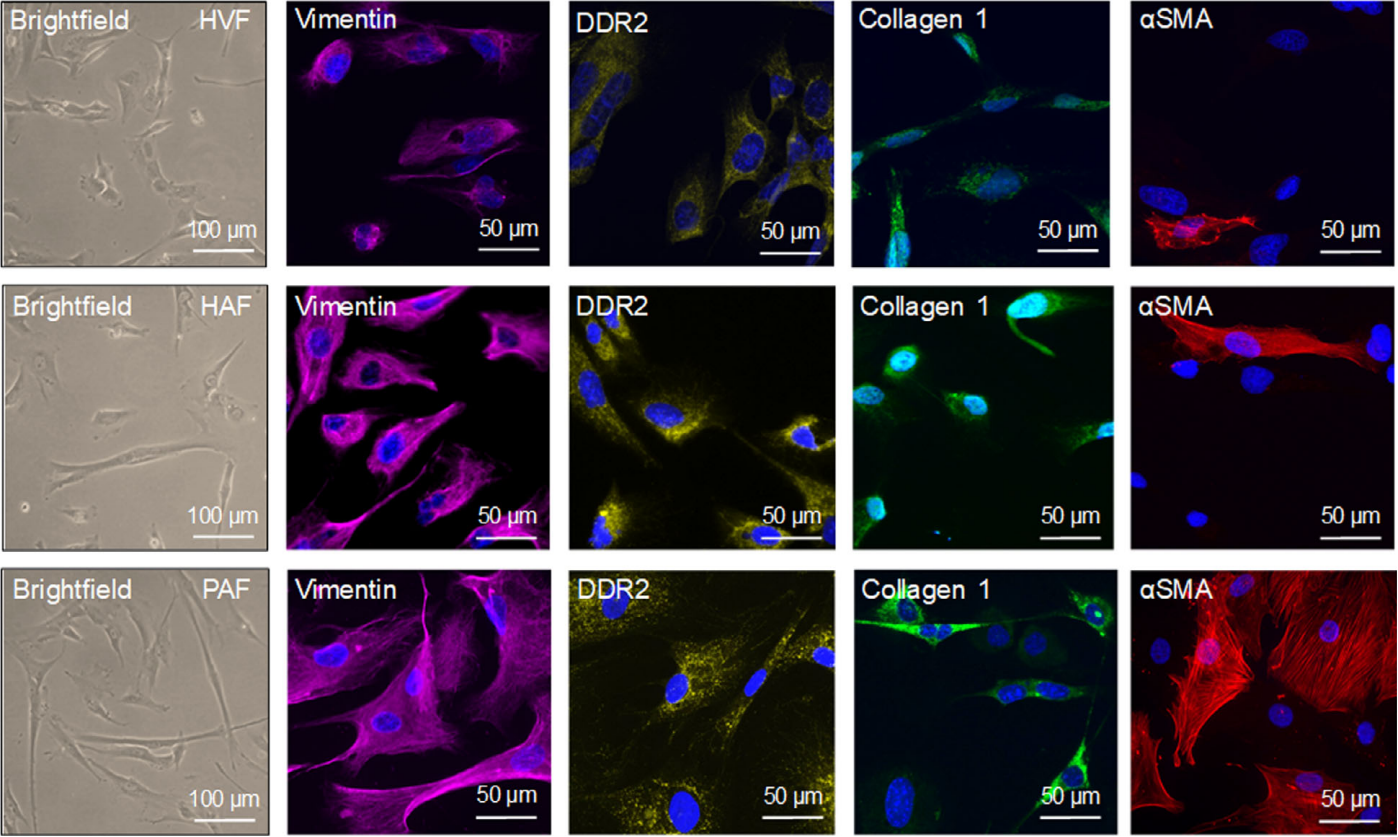
Morphological and immunocytochemical fibroblast identification. Representative brightfield and immunofluorescence images of the fibroblast markers vimentin, DDR2, collagen 1, and αSMA. The nuclei were stained with DAPI (blue). Upper panel) HVFs. Mid panel) HAFs. Lower panel) PAFs. The scale bars equal 50 µm.

Myofibroblast differentiation was assessed with immunocytochemical staining for αSMA in fibroblasts grown on glass coverslips. A cell was considered a myofibroblast when orderly arranged αSMA microfilaments were present (Figs 1 and 4C). A minimum of 50 cells/ coverslip was analyzed. The primary and secondary antibodies used for immunocytochemistry (ICC) are provided below (Table 2).

**Table 2:** Antibodies.

Antibody	Dilution	Conjugate/ Source	Lot‐Nr.	Application
GAPDH	1 : 1000	Mouse	sc‐365062	WB
DDR2	1 : 200	Mouse	MAB25381	ICC
Vimentin	1 : 200	Rabbit	#5741	ICC
αSMA	1 : 200 (ICC) 1 : 1.000 (WB)	Mouse	A5228	ICC/ WB
Collagen 1	1 : 200	Rabbit	ab34710	ICC
Collagen Iα1	1 : 100	Goat	MyBioSource	ICC
SMAD2/3	1 : 1.000	Rabbit	#3102	WB
PhosphoSMAD2/3	1 : 1.000	Rabbit	#8828	WB
Anti‐mouse	1 : 10 000	Peroxidase	A3682	WB
Anti‐rabbit	1 : 10 000	Peroxidase	111‐035‐045	WB
Alexa Fluor 546 (anti‐mouse)	1 : 400	Streptavidin	Z25004	ICC
Alexa Fluor 546 (anti‐rabbit)	1 : 400	Streptavidin	Z25304	ICC
Alexa Flour 555 (anti‐goat)	1 : 500	Rabbit	ab150146	ICC

#### Migration

To evaluate fibroblast migration capacity, a commercially available wound healing assay was performed (Cell Biolabs, BIOCAT GMBH, Heidelberg, Germany) according to the manufacturer’s instructions.

### Collagen secretion and deposition

Soluble collagen secretion was determined in culture medium using a colorimetric hydroxyproline assay kit (Sigma‐Aldrich, St. Louis, Missouri, USA) according to the manufacturer’s instructions. Collagen deposition on the growth surface was visualized using ICC against collagen Iα1. In order to facilitate collagen production, the medium was supplemented with 0.5 mm L‐Ascorbic Acid (AAcid) [16]. AAcid is a necessary cofactor for prolyl and lysyl hydroxylases, enzymes essential for the collagen biosynthesis. Cells were cultured for 6 days.

### Cell stiffness

The effective Young’s modulus, E_Eff_, was assessed using the Chiaro nanoindenter system (Optics11, Amsterdam, Netherlands) as described previously [17]. Briefly, a spherical tip with 3 µm radius, attached to a calibrated cantilever with a spring constant of 0.014 N·m^−1^, is used to indent the sample while the bending of the cantilever is tracked by interferometry. The force required for sample indentation was calculated as the product of cantilever bending and spring constant, and the effective Young’s modulus (E_Eff_) was derived using the Hertzian model for contact mechanics [18] under the assumption of Poisson’s ratio of 0.5 for incompressible materials, which is commonly used for mechanical testing of cells and tissue [19]. Throughout this manuscript, E_Eff_ is referred to as stiffness. On each cell, indentations were performed at three different positions, excluding the nuclear region. Prior to stiffness measurements, cells were cultured on CyPhyGels for 4 days. These are hydrogels based on polyethylene glycol and the cyanobacterial photoreceptor Cph1* [Cph1‐Y263F (amino acids 1–514)] allowing modulation of gel stiffness between 3 and 6 kPa by light [17, 20].

### Statistics

Statistical analysis and graphic representation of the data were done with graphpad prism software (version 5, GraphPad Software, San Diego, CA, USA). Data are presented as mean ± SEM. Differences between two groups were compared using Student’s *t*‐test with Welch’s correction. Differences between multiple groups were compared with one‐way ANOVA with Newman–Keuls post‐test. *P*‐values < 0.05 were considered statistically significant.

### SDS/PAGE, western blotting, and immunodetection

Protein was extracted from fibroblasts for western blot (WB) analysis using RIPA buffer (30 mm Tris, 0.5 mm EDTA, 150 mm NaCl, 1% NP‐40, 0.1% SDS) supplemented with 10% protease and phosphatase inhibitors. The protein concentration was determined using a bicinchoninic acid kit. Subsequently, a standard WB protocol was performed [21].

## Results and Discussion

The scientific value of primary human cells is undisputable although their availability is limited due to ethical and medical reasons. Cell lines have therefore been well established as a valuable alternative. In contrast to primary cells, cell lines do not display interpatient variability due to donor age, medication, or morbidities and theoretically provide an almost unlimited quantity of material. These features are particularly attractive to study cardiac pathomechanisms, since cardiovascular‐patient‐derived primary cells typically display high variability [22]. For this reason, we generated a novel cell culture model of the less commonly available atrial fibroblast subtype to study cardiac fibrosis mechanisms *in vitro*. Subsequently, the newly developed cell line ‘HAF‐SRK01’ was functionally characterized and compared to PAF and commercially available HVF.

**Fig. 2:**
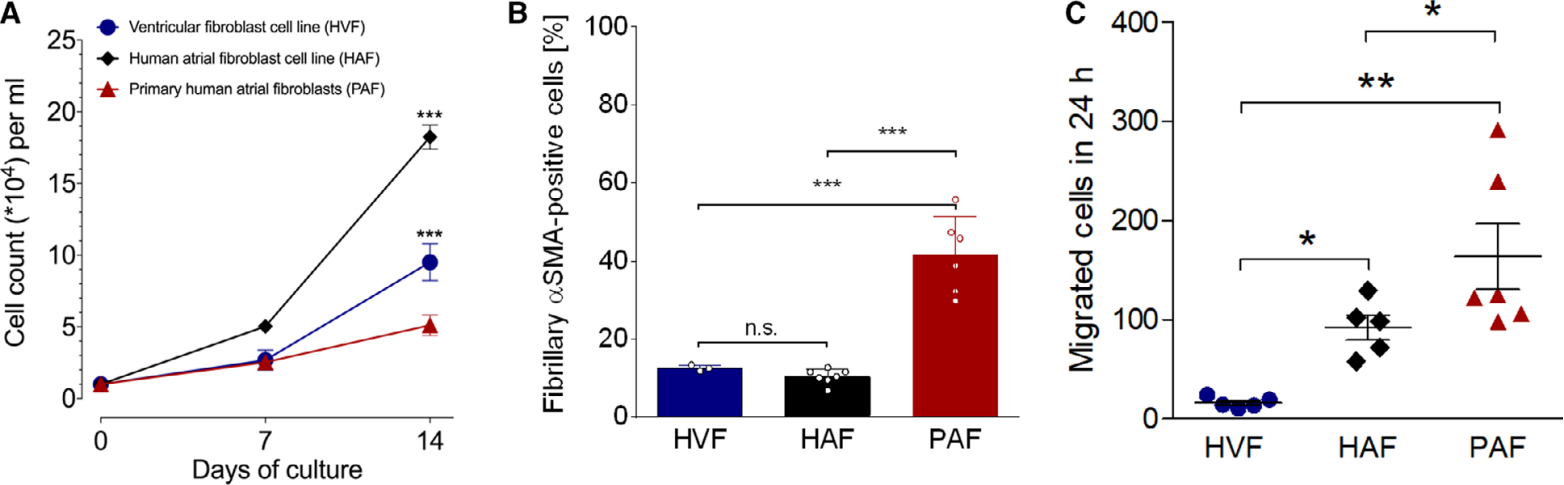
Functional analysis of cardiac fibroblast subtypes. (A) Proliferation curves of HVFs (*n* = 6), HAFs (*n* = 7) and PAFs (*n* = 21). Cells were cultured in DMEM (10% FCS, 1% penicillin–streptomycin) at 37 °C, 5% CO_2_, and counted after 7 and 14 days. (B) Quantification of immunostaining experiments for fibrillary αSMA protein abundance (*n*_HVF_ = 4, *n*_HAF_ = 7, *n*_PAF_ = 6). (C) Basal 24‐h migration capacity of HVFs (*n* = 5), HAFs (*n* = 5), and PAFs (*n* = 6). Data are presented as mean ± SEM. Differences between multiple groups were compared with one‐way ANOVA with Newman–Keuls post‐test. *P < 0.05. **P < 0.01. ***P < 0.001; n.s., not significant.

In brightfield microscopy, HVFs, HAFs, and PAFs displayed the typical stellar fibroblast shape without notable morphological differences between the groups. All cells expressed the accepted fibroblast marker proteins [11] vimentin, discoidin domain receptor 2 (DDR2), and collagen 1 confirming their fibroblast phenotype (Fig. 1). Only the expression of the myofibroblast marker αSMA varied distinctly between the groups (quantification provided in Fig. 2B).

Proliferation and myofibroblast differentiation are essential fibroblast properties that are important for the study of fibrosis mechanisms. Therefore, these parameters were analyzed and compared in HVFs, HAFs, and PAFs. Cell proliferation was strikingly different between the three groups (Fig. 2A). PAFs proliferated at the lowest rate, followed by intermediately proliferating HVFs. The newly generated HAFs displayed the highest proliferation rate (20‐fold increase in cell number compared to day 0). Likewise, spontaneous myofibroblast differentiation as determined by the presence of fibrillary αSMA myofilaments in immunofluorescence assays differed between the groups. HVFs and HAFs displayed comparably low spontaneous differentiation (< 15% of total cells), whereas PAF cultures consisted of ~ 50% myofibroblasts (Fig. 2B). These results point at an inverse correlation of proliferation and myofibroblast differentiation which is consistent with the hypothesis that myofibroblasts are terminally differentiated cells [23, 24] and as such cease to proliferate. These results support recent findings suggesting that fibroblast differentiation rather than proliferation is crucial for fibrosis development [25]. A possible reason for the high spontaneous differentiation and resulting low proliferation rates in PAFs could be patient‐specific imprinting [26] due to donor age, medication, or comorbidities. Epigenetic regulation of proliferation‐relevant genes was shown to affect functional properties of isolated cells [27, 28]. The finding of, for example, aberrant methylation patterns in fibroblasts from AF patients compared to control samples reinforces this assumption [21] and stresses the value of adequate cell lines matching the scientific question.

Fibroblast migration to areas of myocardial tissue damage is important for wound healing and fibrosis development in the heart [29, 30]. We compared the migration capacity of HVFs, HAFs, and PAFs with an *in vitro* wound healing assay [10]. HVFs migrated to the smallest extent followed by HAFs. PAFs displayed the highest migration capacity among the tested cell populations (Fig. 2C). It has been shown that myofibroblasts—once activated—stimulate fibroblast migration *via* chemotactic signals in auto‐ and paracrine manner [31]. This finding is reflected in the here presented results of the migration and differentiation analysis. The cultures with low myofibroblast content (HVFs and HAFs) displayed lower migration rates than PAF cultures, which consisted of ~ 50% of myofibroblasts (Fig. 2B).

**Fig. 3:**
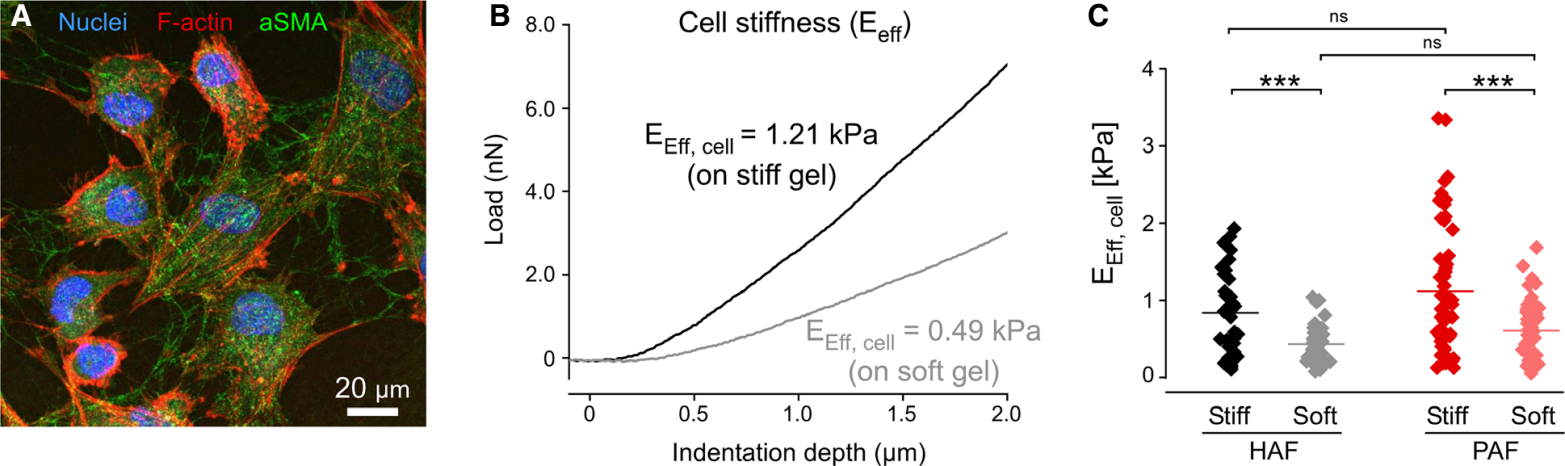
Adaption of HAF and PAF stiffness in response to different stiffness of the growth matrix. (A) HAFs present a typical fibroblast morphology when grown on CyPhyGels. Nuclei were stained with Hoechst (blue), F‐actin was stained with Phalloidin (red), and ɑSMA was stained in green. The scale bar equals 20 µm. (B) Representative force/ indentation curves used to calculate the stiffness (E_eff_) of individual cells cultured on either stiff (black curve) or soft gels (grey curve). The force required to indent a cell on the stiff substrate is higher than on the soft substrate. (C) Measurements of HAF and PAF stiffness on soft (~2.7 kPa) and stiff (~4.6 kPa) CyPhyGels (36 ≤ *n* ≤ 57). Data are presented as mean ± SEM. Differences between multiple groups were compared with one‐way ANOVA with Newman–Keuls post‐test. ***P < 0.001.

An essential feature of fibroblasts is their ability to adapt to the stiffness of their growth matrix. This capability is particularly relevant in the context of fibrosis which leads to a strong increase of tissue stiffness [32, 33]. Stiffening of the growth matrix is well‐known to contribute to the activation of fibroblasts into myofibroblasts and leads to remodeling (stiffening) of the cytoskeleton [17]. We investigated whether HAFs’ response to differences in the stiffness of the growth matrix is preserved by using hydrogels whose stiffness can be controlled by light. HAFs were cultured on CyPhyGels for 4 days (Fig. 3A). Their stiffness was significantly increased on stiff gels compared to soft gels (Fig. 3B,C). HAF stiffness on soft and stiff gels was not different from that of PAFs indicating that the two cell types show comparable adaptations to differences in their mechanical environment (Fig. 3B).

**Fig. 4:**
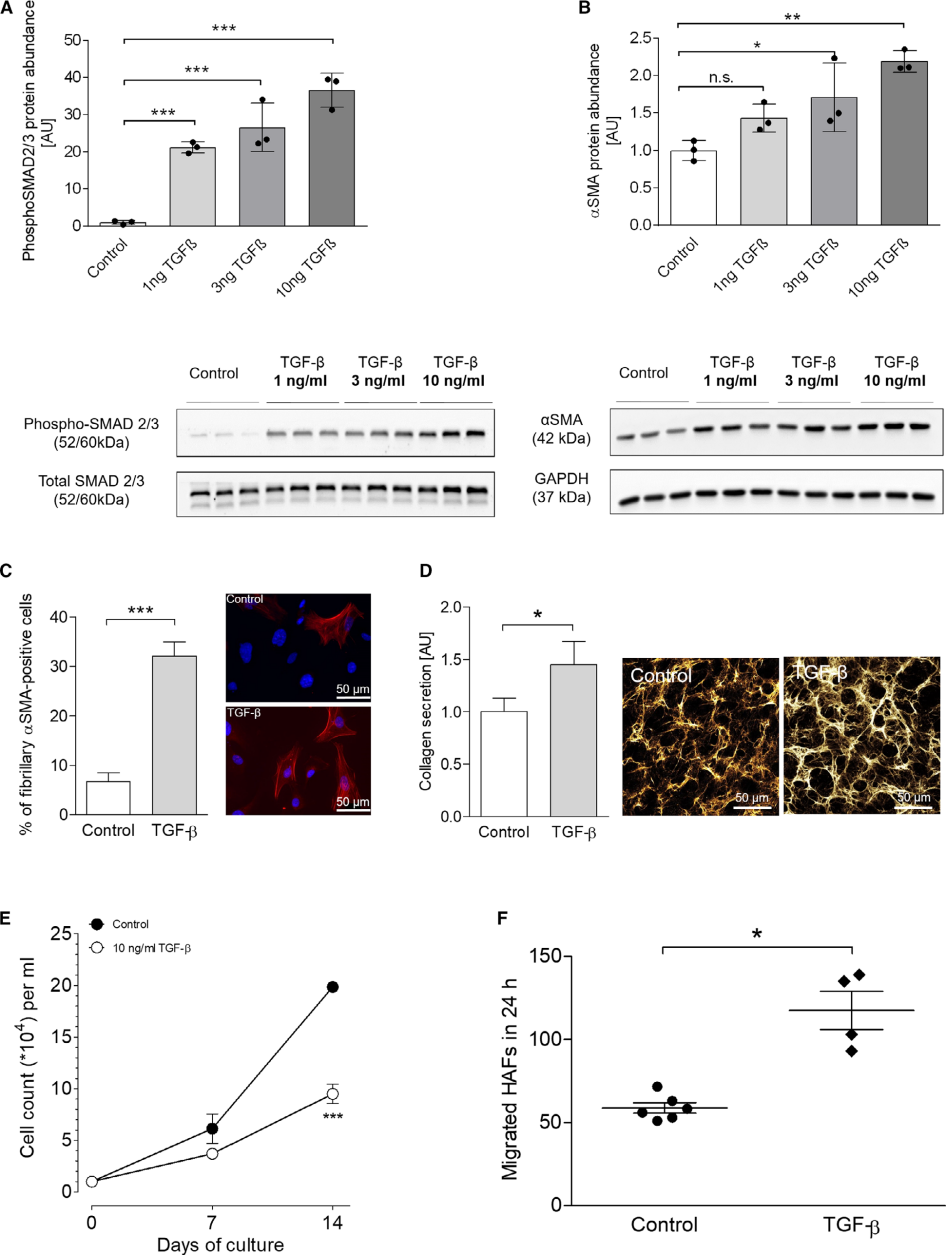
Induction of a cellular fibrosis phenotype in HAFs with TGF‐β. (A) Protein expression of phosphorylated SMAD2/3 after 72 h of TGF‐β stimulation (1, 3, 10 ng·mL^−1^) in HAFs (*n* = 3 per concentration) and representative original WB below. (B) Protein expression of αSMA after 72 h of TGF‐β stimulation (1, 3, 10 ng·mL^−1^) in HAFs (*n* = 3 per concentration) and representative original WB below. (C) Quantification of fibroblasts positive for fibrillary αSMA microfilaments after stimulation with 10 ng·mL^−1^ TGF‐β for 72 h and representative immunofluorescence staining of fibrillary αSMA upon TGF‐β stimulation. The scale bars equal 50 µm. (D) Soluble collagen secretion (left) and representative immunofluorescence image for deposited Collagen Iα1 (right) by HAFs upon stimulation with 10 ng·mL^−1^ TGF‐β (*n* = 14 vs. 10). The scale bars equal 50 µm. (E) Proliferation curves of HAFs under control conditions (*n* = 4) and upon stimulation with 10 ng·mL^−1^ TGF‐β (*n* = 4). Cells were counted after 7 and 14 days. (F) 24‐h migration capacity of HAFs under control conditions (*n* = 6) and upon stimulation with 10 ng·mL^−1^ TGF‐β (*n* = 4). Data are presented as mean ± SEM. Differences between two groups were compared using Student’s *t*‐test with Welch’s correction. Differences between multiple groups were compared with one‐way ANOVA with Newman–Keuls post‐test. *P < 0.05. **P < 0.01. ***P < 0.001.

One key mechanism in fibrosis development is the activation of the TGF‐β‐SMAD pathway [34]. TGF‐β is considered the master regulator of fibroblast activation and fibrosis [35]. It is therefore important that a novel *in vitro* fibroblast model responds adequately to stimulation with TGF‐β. We exposed the HAF‐SRK01 cell line to TGF‐β in ascending concentrations (1, 3, 10 ng·mL^−1^) for 72 h. HAFs responded to TGF‐β with a concentration‐dependent increase in SMAD2/3 phosphorylation and concomitant increase in αSMA protein expression (Fig. 4A,B) as determined by WB [36]. Immunofluorescence revealed the increased presence of fibrillary αSMA myofilaments confirming myofibroblast differentiation (Fig. 4C). Furthermore, we investigated collagen secretion, which is the crucial step of structural ECM remodeling and fibrosis development. HAFs responded with increased collagen secretion and deposition in response to TGF‐β stimulation (Fig. 4D). Finally, we investigated the effects of TGF‐β on HAF proliferation and migration. We found significantly reduced fibroblast proliferation after 14 days (Fig. 4E) and significantly increased cell migration (Fig. 4F). These results are consistent with the hypothesis that myofibroblasts proliferate less but show increased migration capacity [23, 24]. Taken together, these results demonstrate the functionality of the cell line as an *in vitro* fibrosis model as it is able to recapitulate the essential cellular steps and mechanisms in the development of fibrosis.

There are limitations to the current study, which should be addressed in future research on this topic. Since we had only limited access to atrial appendage samples from control (sinus rhythm) patients, the group size of some experiments depending on PAFs is limited. It must also be considered that control fibroblasts do not originate from healthy donors since the patients had to undergo cardiac surgery as well. Despite these limitations, we are convinced that our newly generated cell line is a valuable addition to the existing spectrum of methods for cardiac fibrosis research.

With low basal myofibroblast differentiation, high proliferation and migration rates and conserved mechano‐sensing (adaptation to their mechanical environment), our newly generated HAF cell line HAF‐SRK01 can serve as a robust *in vitro* model to improve the understanding of atrial fibrosis mechanisms. This model can effectively complement research on PAFs, as it is continuously available on a large scale due to high proliferation rates, making its use more flexible and most importantly independent of patient or animal sample acquisition. Moreover, our study emphasizes that cardiac fibroblast subtypes are phenotypically heterogeneous with distinct functional differences depending on their ventricular or atrial origin. Analyzing the proliferative, migratory, and spontaneous myofibroblast differentiation capacity, we show that patient‐specific variations have a major influence on the results obtained with primary cells. Accordingly, PAFs displayed the highest basal myofibroblast differentiation stressing the fact that the proper choice of a fibroblast model is critical to avoid misinterpretations, as the causes of variability, for example, in myofibroblast differentiation (e.g., donor medication and comorbidities) cannot always be determined retrospectively. Even though cell lines have limitations on their own, they offer the advantage of more consistent results due to the lack of patient‐related variability. This and the fact that no common cell line model of atrial fibroblasts currently exists make the HAF‐SRK01 line particularly interesting for mechanistic questions and screening experiments. We expect that our new HAF cell line will become an increasingly useful model to study fibrosis *in vitro*, as there is an urgent need for effective antifibrotic pharmacotherapy, especially in the context of AF. Improved understanding of atrial fibrosis mechanisms will open up new opportunities for disease modeling and chamber‐specific drug screening.

## Conflict of interest

The authors declare no conflict of interest.

## Author contributions

SRK conceived and supervised the study. SRK, EK, AEA, KL, UR, JSER, EARZ, and RP designed experiments. JSER, KK, CS, MH, TK, SD, RE, EARZ, and RP performed experiments. NH and JHK immortalized primary donor cells. SRK wrote the manuscript and all authors revised and improved the manuscript. Parts of SRK’s, JSER’s, MH’s, and CS’s doctoral theses are included in this study.
